# Antibacterial Properties of the Mammalian L-Amino Acid Oxidase IL4I1

**DOI:** 10.1371/journal.pone.0054589

**Published:** 2013-01-23

**Authors:** Marie-Line Puiffe, Isabelle Lachaise, Valérie Molinier-Frenkel, Flavia Castellano

**Affiliations:** 1 INSERM, U955, IMRB, Equipe 09, Créteil, France; 2 Université Paris Est, Faculté de Médecine, Créteil, France; 3 AP-HP, Hôpital H. Mondor - A. Chenevier, Service d’Immunologie Biologique, Créteil, France; 4 Plateforme Chromatographie Analytique et semi Préparative, ICMPE, Thiais, France; Instituto Butantan, Brazil

## Abstract

L-amino acid oxidases (LAAO) are flavoproteins that catalyze the oxidative deamination of L-amino acids to a keto-acid along with the production of H_2_O_2_ and ammonia. Interleukin 4 induced gene 1 (IL4I1) is a secreted LAAO expressed by macrophages and dendritic cells stimulated by microbial derived products or interferons, which is endowed with immunoregulatory properties. It is the first LAAO described in mammalian innate immune cells. In this work, we show that this enzyme blocks the *in vitro* and *in vivo* growth of Gram negative and Gram positive bacteria. This antibiotic effect is primarily mediated by H_2_O_2_ production but is amplified by basification of the medium due to the accumulation of ammonia. The depletion of phenylalanine (the primary amino acid catabolized by IL4I1) may also participate in the *in vivo* inhibition of staphylococci growth. Thus, IL4I1 plays a distinct role compared to other antibacterial enzymes produced by mononuclear phagocytes.

## Introduction

L-amino acid oxidases (LAAO) are homodimeric flavoproteins that catalyse the stereospecific deamination of L-amino acid substrates to a keto-acid along with the production of H_2_O_2_ and ammonia. These enzymes are widely expressed in many different organisms from prokaryotes to metazoans, of which snake venom LAAO being the most studied. Their function is still poorly understood. Venom LAAO have been suggested to act as toxins involved in the induction of apoptosis in a variety of different mammalian cell types. In addition, they are associated with the dysfunction of platelet aggregation [1].

Interleukin 4 induced gene 1 (IL4I1) is a secreted mammalian LAAO primarily expressed by activated mononuclear phagocytes, such as macrophages and dendritic cells, under the influence pro-inflammatory and T helper type 1 (Th1) mediators *in vitro*. Accordingly, IL4I1 is highly expressed in Th1 but not in Th2 granulomas. This enzyme has also been detected in B lymphocytes following activation by IL-4 and CD40, although at much lower levels [2]. At physiological pH and temperature, IL4I1 degrades the essential amino acid phenylalanine (Phe), producing phenylpyruvate, H_2_O_2_ and ammonia. By this activity, IL4I1 depletes the microenvironment of an essential amino acid and induces the accumulation of potentially toxic products. We have demonstrated that IL4I1 is involved in the control of the adaptative immune response *via* its enzymatic activity [3], [4].

Because of H_2_O_2_-dependent cytotoxic effects and the potential toxicity of other resulting catabolites, LAAO family members may play a variety of roles in immune defense in animals. A snake venom LAAO has been shown to present potent antibacterial activities against Gram-positive and Gram-negative bacteria which is related to H_2_O_2_ production [5]. An LAAO with protective activity against bacterial mastitis has also been detected in mouse milk [6]. IL4I1 is phylogenetically related to fish LAAO [7], which have been shown to present antibacterial functions [8] and accumulate in “granuloma-like” structures induced by the infection with larval nematodes [9]. Recently, a new fish LAAO has been described, which may contribute to the innate immune defense against a variety of bacteria and protozoans [10]. As IL4I1 expression is induced in mononuclear phagocytes by pathogen-derived signals, such as Toll-like receptor ligands [2], it may participate in the innate immune defense against pathogens in mammals, in addition to its regulatory effects on the specific immune response.In this work, we thus evaluated the effect of recombinant IL4I1 on Gram-positive and Gram-negative bacteria growth. We show that IL4I1 can act as an antibiotic, which could potentially contribute to the arsenal used by activated macrophages to generate an extracellular environment hostile to bacterial growth.

## Results

**Figure 1:**
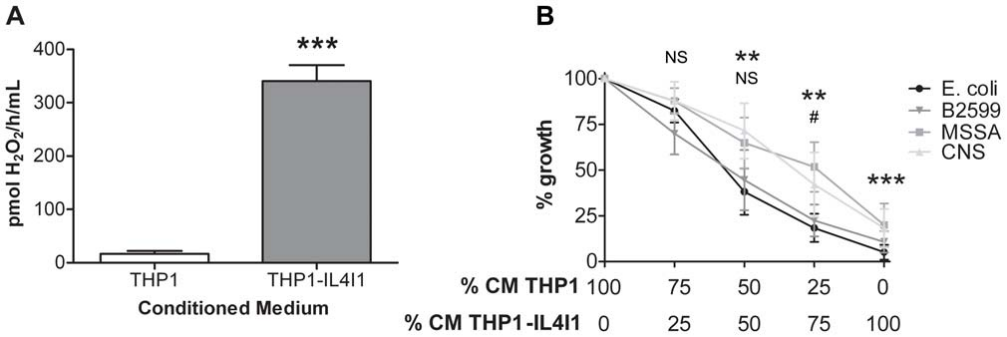
IL4I1 inhibitory growth effect. (**A**) IL4I1 activity in conditioned medium from THP1 and THP1-IL4I1 cells (0.5×10^6^ cells/ml cultured in DMEM/F12+1% FCS, 48 hours at 37°C). Results are expressed as pmol H_2_O_2_ produced in the presence of phenylalanine per hour per ml of conditioned medium. (**B**) Bacteria were diluted in conditioned medium (CM) from THP1 containing different ratios of IL4I1-conditioned medium. After 24 hours, bacterial growth was monitored at an OD of 595 nm. Data are given as mean ± SEM from five independent experiments performed in duplicate. Mean OD in 100% CM THP1 was 0.227 for *E. coli*, 0.266 for B2599, 0.450 for MSSA and 0.516 CNS, respectively. These OD were considered as 100% bacterial growth and the OD measured in the other culture conditions were calculated as percentages with respect to this control. Statistical analyses were performed using the Mann-Whitney test in comparison to 100% CM THP1. ***p<0.001 for all the bacterial strains at 100% CM THP1-IL4I1; **p<0.01 for *E. coli*, B2599 and MSSA and ^#^ *p*<0.05 for CNS at 75%; **p<0.01 for *E. coli* and B2599 at 50%; NS, not significant for MSSA and CNS at 50% and for all the strains at 25%.

We tested the antibacterial effect of conditioned media from IL4I1-expressing THP1 monocytes on four bacterial strains, representative of Gram-negative bacilli and Gram-positive cocci, i.e. *Escherichia coli* DH5α (*E. coli*), an *Escherichia coli* strain auxotrophic for Phe (B2599) and 2 *Staphylococcus* strains, a methicillin-susceptible *Staphylococcus aureus* (MSSA) and a coagulase-negative *Staphylococcus* (CNS). IL4I1 activity in these media was 340.88 pmol/H_2_O_2_/h/ml ±66.27 (**Figure 1A**). The IL4I1-containing medium was then diluted in control-conditioned medium from THP1 cells at increasing ratios (**Figure 1B**). We observed that the growth of all four bacterial strains was blocked in an IL4I1 dose-dependent fashion, indicating an antibacterial effect of the enzyme.

IL4I1 degrades L-Phe and to a lesser extent L-tryptophan (Trp) to the corresponding α-keto-aminoacid, NH_3_ and H_2_O_2_. The activity of IL4I1 is usually measured based on H_2_O_2_ production in the presence of Phe (**Figure 1A**). Since all the catabolites of the reaction are theoretically produced in equimolar amounts, we expected ammonia accumulation in the presence of IL4I1. Although IL4I1-mediated NH_3_ production could not be detected in THP1 and THP1-IL4I1 conditioned media, because of a strong basal level mainly due to glutamine instability, we confirmed the production of ammonia in Phe-containing conditioned PBS (**Figure S1**). The 24 h accumulation of ammonia in THP1 cultures (74.0±16.2 µM) doubled in the presence of IL4I1 (153.8±27.6 µM). Moreover, the presence of IL4I1 induced a 0.5 pH increase in the medium (THP1: pH = 7.5; THP1-IL4I1: pH = 8) that was not modified by bacterial growth. The Phe, Trp and phenylpyruvate content were measured using an HPLC technique. Similar to NH_3_, phenylpyruvate was significantly increased in the presence of IL4I1 (**Figure S2**). Phe and Trp content were at the limit of sensitivity of the method. Two samples were transferred to a laboratory specialized in Phe quantification in human serum and urine. No significant variation in Phe content was measured, as expected from the low rate of Phe consumption (less then 10 µM/24 h).

**Figure 2:**
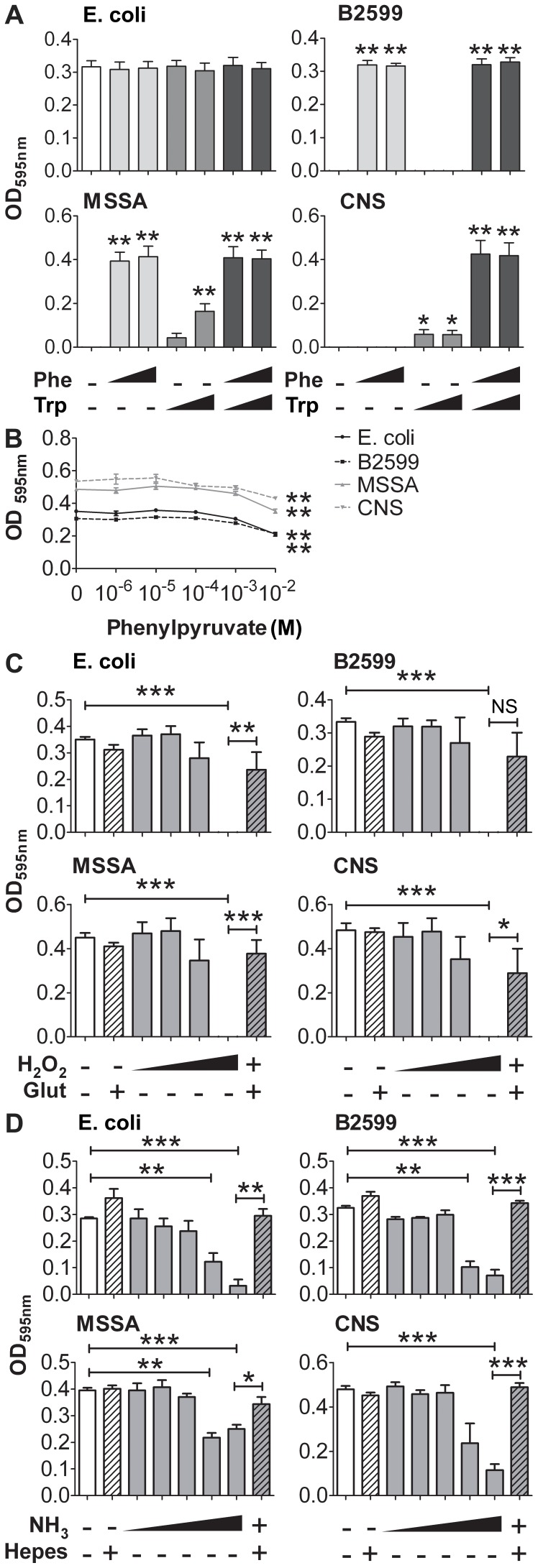
Susceptibility of bacteria to IL4I1 substrates and catabolites. Bacteria were serially diluted in DMEM/F12, with: (**A**) no Phe, 35.49 or 500 µg/ml Phe and/or no Trp, 9.02 or 500 µg/ml Trp; (**B**) ten folds dilutions of phenylpyruvate from 1 µM to 10 mM; (**C**) ten folds dilutions of H_2_O_2_ from 1 µM to 1 mM or 64 µg/ml glutathione with or without 1 mM H_2_O_2_; (**D**) ten folds dilutions of NH_3_ from 5 µM to 50 mM or 15 mM HEPES with or without 50 mM NH_3_. After 24 hours, bacterial growth was monitored at an OD of 595 nm. Data are given as mean ± SEM from five independent experiments performed in duplicate. *p<0.05 **p<0.01 ***p<0.001, Mann-Whitney test in comparison to DMEM/F12 without Phe and Trp (**A**), without phenylpyruvate (**B**) or according to the bars on the graph (**C** and **D**).

We sought to determine which mechanisms associated with this reaction could be involved in the observed antibacterial effect (**Figure 2**). We first explored the role of amino acid consumption in the bacterial microenvironment. For this purpose, bacteria were grown in DMEM/F12 supplemented with glutathione but lacking Phe and Trp. Both amino acids were alternatively added to the medium, alone or in combination (**Figure 2A**). *E. coli* growth was not affected by amino acid deprivation. In contrast, the growth of the three other strains was blocked in Phe- and Trp-deprived media. As expected, the growth of B2599 was restored by Phe, but not by Trp addition. The presence of Trp in *Staphylococcus* cultures only partially restored their growth. However, MSSA could grow in Trp-depleted medium if Phe was present, while CNS growth was only restored by the presence of both amino acids. Thus, these three strains were variably auxotrophic for Phe and Trp.

We next explored the role of the three catabolites of the enzymatic reaction (**Figure 2B, C, D**). Phenylpyruvate was weakly toxic at high concentrations (10 mM). In contrast, H_2_O_2_ totally inhibited the growth of the four bacterial strains at 1 mM, despite their endogenous catalase production. This effect was abolished by the addition of glutathione in the culture media. Ammonium (NH4^+^) is considered as a source of nitrogen for bacterial growth. However, addition of the basic compound ammonia (NH_3_) to the *E. coli* and staphylococci cultures displayed a toxic effect at doses ≥ 5 mM, which was reverted by buffering the media with HEPES.

**Figure 3:**
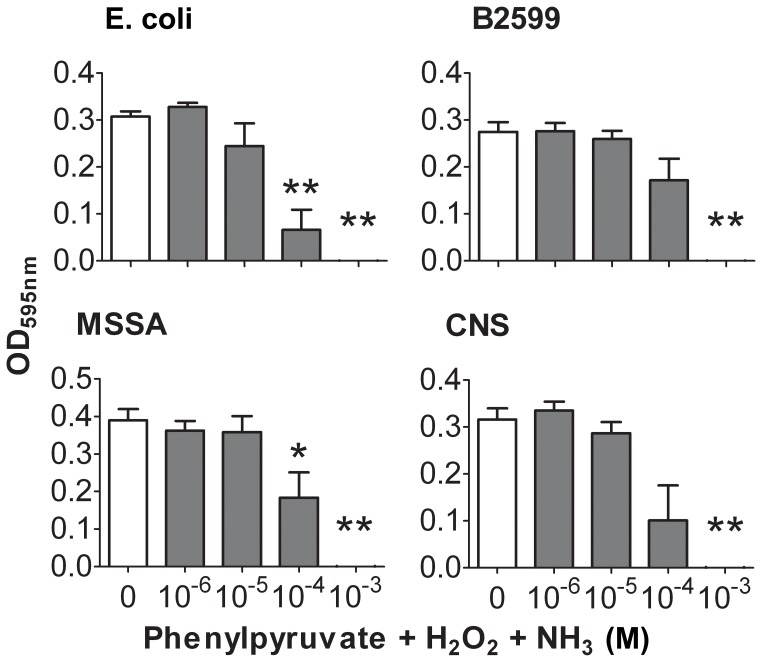
Susceptibility of bacteria to the catabolite mix. Bacteria were serially diluted in RPMI 1640 with or without isomolar addition of phenylpyruvate, H_2_O_2_ and NH_3_ from 1 µM to 1 mM. After 24 hours, bacterial growth was monitored at an OD of 595 nm. Data are given as mean ± SEM from five independent experiments performed in duplicate. **p<0.01 and *p<0.05, Mann-Whitney test in comparison to RPMI 1640.

We verified if combining all three IL4I1 catabolites could amplify their individual effect. Isomolar amounts of phenylpyruvate, H_2_O_2_ and NH_3_ were added to a culture medium lacking HEPES but containing minimal amounts of glutathione, Trp and Phe (**Figure 3**). Under these conditions, the growth of *E.coli* and MSSA was sensitive to doses 10 times lower than when H_2_O_2_ was used alone. A similar effect was obtained by mixing H_2_O_2_ and NH_3_ only (**Figure S3**). Moreover, the inhibition of bacterial growth was lost in a HEPES-buffered medium containing the three catabolites (**Figure S4**), suggesting that NH_3_ potentiates the toxic effect of H_2_O_2_ *via* basification of the medium. This was confirmed using NaOH as basificating agent. Indeed, *E coli* was sensitive to ten times less H_2_O_2_ when the pH was increased from 7.5 to 8.5, an increase that can also be obtained with 5 mM NH_3_ and that is not toxic by itself (**Figure S5**). In summary, both amino acid depletion and the presence of phenylpyruvate, H_2_O_2_ or NH_3_ affected, to variable degrees, bacterial growth (**Table 1**). Moreover, the presence of equimolar quantities of H_2_O_2_ and NH_3_ from the IL4I1 reaction could have a cumulative effect, indicating that their simultaneous production by the enzyme may amplify its antibacterial properties.

**Table 1:** Effect of amino acid depletion and IL4I1 catabolites on bacterial growth.

	Amino acid depletion			IL4I1 catabolites addition			
	Phe	Trp	Phe & Trp	PP	H_2_O_2_	NH_3_	PP+H_2_O_2_+NH_3_
***E. coli***	No effect	No effect	No effect	10 mM	1 mM	5 mM	0.1 mM
**B2599**	Inhibition	No effect	Inhibition	10 mM	1 mM	5 mM	1 mM
**MSSA**	Partial inhibition	No effect	Inhibition	10 mM	1 mM	5 mM	0.1 mM
**CNS**	Partial inhibition	Inhibition	Inhibition	10 mM	1 mM	50 mM	1 mM

Effect of Phe and Trp substrate deprivation and molar concentration H_2_O_2_, NH_3_ and phenylpyruvate (PP) addition to liquid cultures of Gram^–^ (*E. coli* and B2599) and Gram^+^ (MSSA and CNS) bacteria.

**Figure 4:**
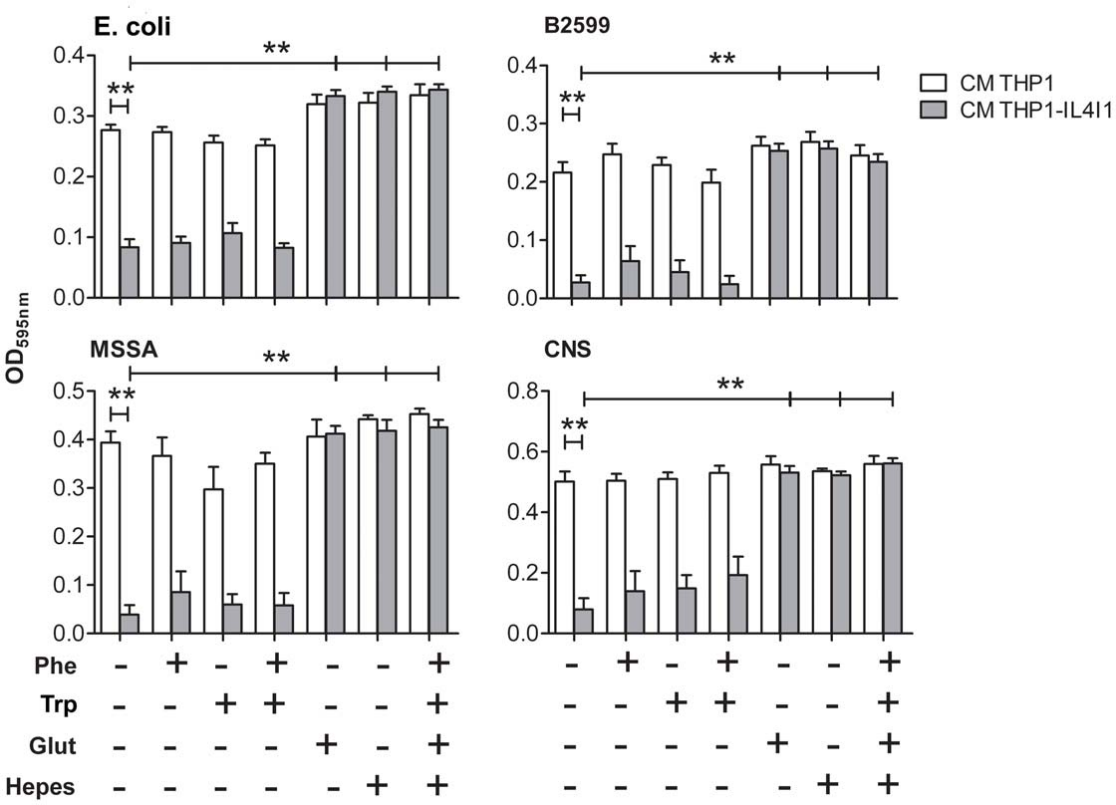
Rescue from IL4I1-induced bacterial growth inhibition. Bacteria were serially diluted in conditioned medium of THP1 or THP1-IL4I1 cells supplemented with 500 µg/ml Phe, 500 µg/ml Trp or both, 64 µg/ml glutathione or 15 mM HEPES or the combination of the four reagents. After 24 hours, bacterial growth was monitored at an OD of 595 nm. Data are given as mean ± SEM from five independent experiments performed in duplicate. **p<0.01, Mann-Whitney test in comparison to growth in THP1-IL4I1 conditioned medium without reagent addition.

In order to confirm these putative mechanisms of IL4I1 action, we added Phe, Trp, glutathione, and HEPES, either separately or in combination, to bacterial cultures grown in conditioned media containing or not IL4I1. While IL4I1 was still toxic for the bacteria after the addition of a large excess (0.5 mg/ml) of Phe or Trp or both, either H_2_O_2_ scavenging with glutathione or NH_3_ buffering with HEPES were sufficient to restore full growth of *E. coli* and staphylococci (**Figure 4**). This data suggest that, under our experimental conditions, the antibiotic action of IL4I1 is mainly due to H_2_O_2_ and NH_3_ production whereas its LAAO activity does not sufficiently deplete the medium of Phe or Trp to inhibit bacterial growth, as indicated by HPLC analysis.

**Figure 5:**
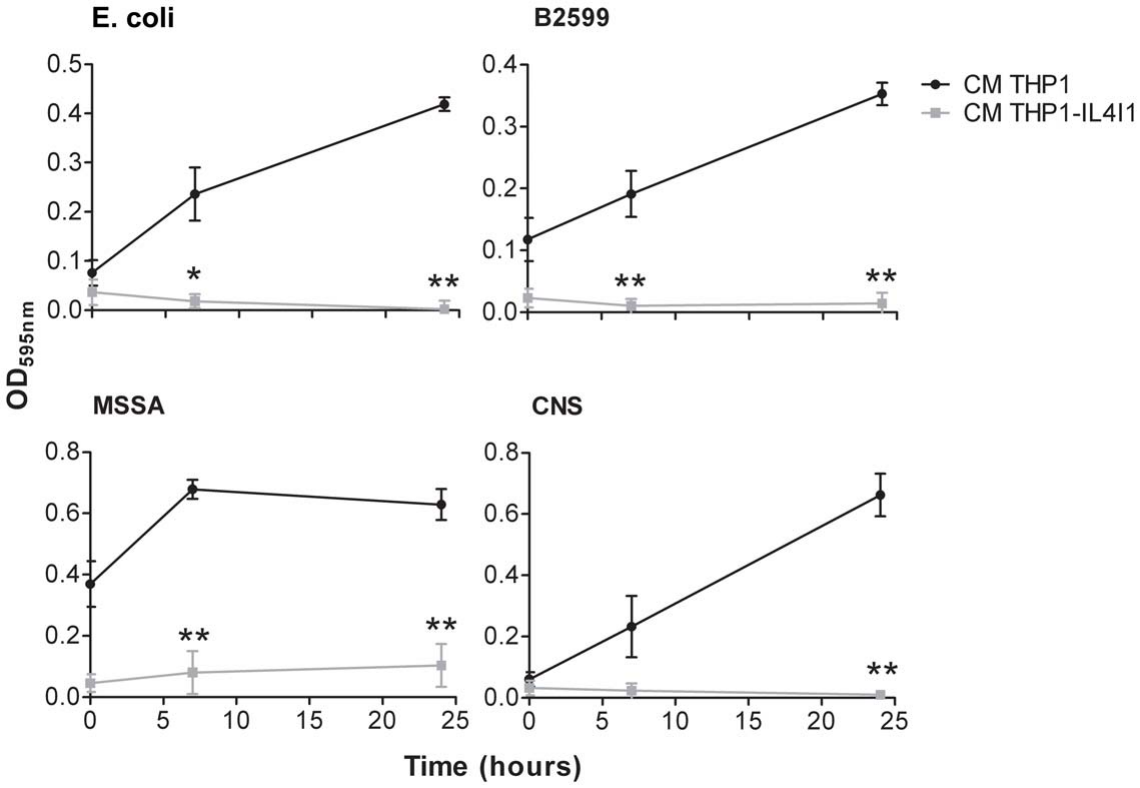
IL4I1 bactericidal effect. Bacteria were grown 24 hours in serial dilutions of conditioned medium of THP1 or THP1-IL4I1 cells and OD measured. The lowest OD for both conditions was selected (t = 0). New DMEM/F12 containing 1% FCS was added, then bacterial re-growth was measured after 7 and 24 hours and compared between bacteria pre-cultured in THP1 and THP1-IL4I1 conditioned medium. Data are given as mean ± SEM from five independent experiments performed in duplicate. *p<0.05 and **p<0.01, Mann-Whitney test.

The antibiotic effect of IL4I1 might be due to killing of the bacteria or simply to the blockade of their growth. To analyze this aspect, bacteria grown in conditioned media were reseeded in fresh medium without IL4I1. Whereas bacteria preincubated in THP1 control medium displayed vigorous growth in fresh medium, the growth of IL4I1-preincubated bacteria could not be restored under the same conditions (**Figure 5**). This result indicates that IL4I1 is bactericidal.

**Figure 6:**
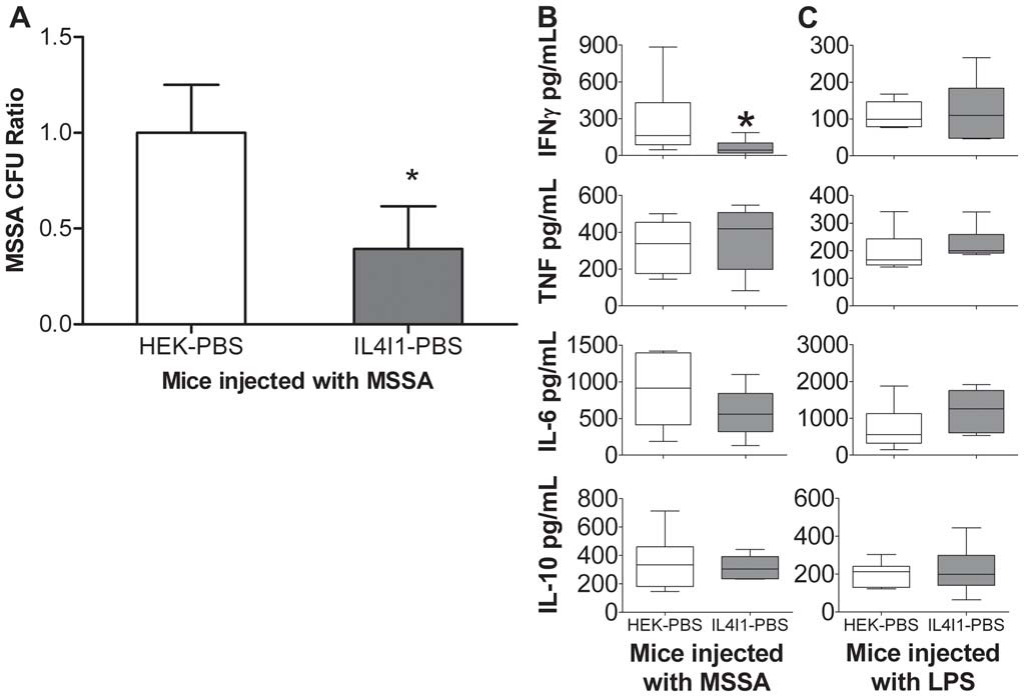
IL4I1 inhibition of bacterial growth *in* *vivo* and associated plasmatic cytokine variations. (**A**) MSSA was added to HEK-PBS or IL4I1-PBS, and the mixes were injected intraperitoneally into groups of three C57Bl/6 mice (mean of 1.74×10^8^ CFU/mouse). Twenty-four hours after injection, supernatants from dissociated spleens were serially diluted and inoculated onto LB agar plates. Bacterial colonies were then counted. The ratio of the CFU in each mouse, relative to the mean CFU from triplicate HEK-PBS mice from each of the four experiments was calculated. Data are represented as mean ratio ± SEM. (**B-C**) Interferon γ (IFNγ), tumor necrosis factor α (TNF), interleukin 6 (IL-6) and interleukin 10 (IL-10) were measured by ELISA in diluted plasma samples from groups of three mice injected with MSSA (**B**), or with 20 µg LPS (**C**). ELISA results are given as mean ± SEM from three experiments. *p<0.05, Mann-Whitney test between the 2 groups.

We finally measured *in vivo* the antibacterial properties of IL4I1. A mean of 1.75×10^8^ CFU of MSSA resuspended in conditioned PBS containing or not the recombinant murine enzyme (specific activity 7.327 nmol/H_2_O_2_/h/ml ±1.518) were injected into the peritoneum of C57Bl/6 mice. For this purpose, we used supernatants from HEK293 cells, which produce the murine form of the enzyme, thus avoiding a potential immune reaction to the human form. In addition, these cells are very resistant when cultured in PBS without serum. Twenty-four hours later, mice were sacrificed and the spleens and blood collected. A significant decrease in the number of bacteria present in the spleen was observed in mice who had received the IL4I1-PBS-MSSA suspension compared to mice injected with MSSA in HEK-PBS (**Figure 6A**). This diminution was accompanied by significantly lower levels of plasma IFNγ and a trend towards lower levels of the proinflammatory cytokine IL-6 (**Figure 6B**). We did not detect significant differences in TNFα and IL-10 concentrations, the level of the latter being identical to those measured in the blood of naïve mice (**Figure S6**). As the cytokines were measured 24 h after bacterial injection, this may be due to the kinetics of cytokine production after an acute infection [11]. These results indicate that IL4I1 could protect against bacterial growth *in vivo*. However, we have previously described IL4I1 as an immunoregulatory enzyme, which inhibits IFNγ production by lymphocytes [2]. We thus verified that the diminution of the IFNγ levels in the sera of mice receiving IL4I1 was not due to a direct effect of the enzyme on IFNγ-producing cells. Mice were thus injected with lipopolysaccharide (LPS) along with IL4I1-PBS or HEK-PBS and cytokines were measured in the plasma at 24 h, while splenocytes were analyzed by flow cytometry for IFNγ production. Under these conditions, no difference in cytokine levels was observed between the two groups of mice (**Figure 6C**). The IFNγ-producing cells were represented by T lymphocytes and NK cells. In all mice that were analyzed, these cells represented 14.6±5.1% of all T lymphocytes and 34.3±7.2% of all NK cells, respectively, regardless of the presence of IL4I1 (representative result in **Figure S7**). Thus, the diminution of plasma IFNγ in mice challenged with bacteria and IL4I1 probably reflects the reduced inflammation associated with the control of the infection.

## Discussion

In this paper, we demonstrate that the phenylalanine oxidase IL4I1 is a bactericidal enzyme, which acts primarily through the production of toxic levels of H_2_O_2_ and NH_3_. This antibacterial effect was observed on both Gram^+^ and Gram^-^ bacteria.

IL4I1 catalyses the oxidative deamination of Phe and to lesser extent Trp thus producing equimolar amounts of an α-ketoacid (phenylpyruvate), H_2_O_2_ and NH_3_ [3]. The well-known toxic effect of H_2_O_2_ was potentiated by basification of the medium by NH_3_, demonstrating that the IL4I1 antibacterial effect does not simply rely on H_2_O_2_ production. Phe or Trp depletion might also participate to growth inhibition in bacterial strains auxotrophic for these amino acids. However, it did not appear to be a major mechanism of action in our *in vitro* experimental conditions, where no diminution of the Phe content could be evidenced. IL4I1 is produced by mononuclear phagocytes stimulated by bacterial products and pro-inflammatory cytokines, such as type I IFN, IFNγ and TNFα [2]. In the context of bacterial infections, IL4I1 could be either secreted at the contact zone between the phagocytic cell and the bacteria, in the recently called “phagosomal synapse” [12] or released in the phagolysosome, in both cases contributing to the bactericidal arsenal of the macrophage.

Several amino acid degrading enzymes, produced by myeloid cells in mammals, have been demonstrated to participate in anti-infectious effects together with an immunosuppressive activity directed towards T lymphocytes [13]. These enzymes share a common mechanism of action: amino-acid depletion together with the production of a variety of toxic compounds, constituting a repertoire of weapons against a large spectrum of diverse microbial targets. The redundancy of this system may also suggest its importance for the host response. IL4I1 thus represents a new member of this complex and coordinated antimicrobial system.

Addition of IL4I1 to bacteria injected in mice diminished the bacterial load in the spleen, concomitantly reducing the inflammatory response, independently of the previously demonstrated immunomodulatory effect of IL4I1. In humans, we previously reported a high level of IL4I1 production in macrophages associated with tuberculosis granuloma [2], suggesting a role for IL4I1 in both the containment of the bacterial dissemination and modulation of the Th1 cell response, in order to preserve the organ from the consequences of uncontrolled inflammation. Such a mechanism has also been proposed for the indoleamine 2,3 dioxygenase enzyme in the context of listeria granuloma [14].

IL4I1 is phylogenetically derived from bony fishes LAAO [7], some of which have been shown to use the enzyme to limit the growth of parasite larvae in structures resembling granuloma [9]. Thus, as it has been described for other aminoacid-catabolising enzymes [13], IL4I1 may have evolved from ancestral innate anti-microbial functions to acquire a regulatory effect on the adaptive immune system.

## Materials and Methods

### Cell Culture, Media and Reagents

Monocytic THP1 and Human embryonic kidney 293 (HEK) cell lines were cultivated respectively in RPMI 1640 and DMEM (Life technologies, Saint Aubin, France) containing 10% FCS (PAA, les Mureaux, France), 2 mM L-glutamine, 100 UI/ml penicillin, 100 µg/ml streptomycin, and for THP1 1 mM sodium pyruvate (all from Life Technologies). Stable THP1 cell lines expressing human IL4I1 (THP1-IL4I1) were obtained as described in Marquet et al [2]. Stable HEK cell lines expressing murine IL4I1 (HEK-mIL4I1) were obtained as described in Boulland et al [3]. Other media used were DMEM/F12 (1∶1) without Phe and Trp (Clinisciences, Montrouge, France). Phe and Trp from Clinisciences were used at concentrations starting from those found in RPMI 1640. Glutathione, HEPES, hydrogen peroxide (H_2_O_2_) and phenylpyruvate were from Sigma Aldrich (Lyon, France), and ammonia (NH_3_) from Honeywell Riedel-de Haën (Seelze, Germany). PBS, Amplex Ultra Red and LB agar were obtained from Life Technologies.

### Preparation of Conditioned Medium

THP1 and THP1-IL4I1 were cultured in DMEM/F12 containing 1% FCS (0.5×10^6^cells/ml) for 2 days and the supernatants used as culture medium for bacteria, in the presence or absence of additional Phe (0.5 mg/ml) and Trp (0.5 mg/ml), glutathione (64 µg/ml) and HEPES (15 mM). HEK-mIL4I1 and HEK transfected with the empty vector were cultured in PBS with calcium and magnesium for 24 hours and the resulting supernatants were used for mouse injections (conditioned PBS from HEK cells defined HEK-PBS and conditioned PBS from HEK-mIL4I1 cells, IL4I1-PBS).

### Enzymatic Activity Measurement

IL4I1 activity was measured in conditioned medium from THP1, THP1-IL4I1, HEK and HEK-mIL4I1. The enzymatic assay was performed as described in Carbonnelle-Puscian et al. [15] using Amplex Ultra Red and expressed as pmol H_2_O_2_ produced in the presence of Phe per hour per ml of conditioned medium.

### Determination of Bacterial Growth

For the analysis of bacterial growth in conditioned medium, *Escherichia coli* (*E. coli*) and *E. coli* B2599 (Phe auxotroph bacteria) from Life Technologies and ASAP (A Systematic Annotation Package for community analysis of genomes, University of Wisconsin), respectively, were used. Methicillin-susceptible *Staphylococcus aureus* (MSSA) and Coagulase negative *Staphylococcus* (CNS) were obtained from routine diagnostic specimens. Bacteria were grown on LB agar. For use in experiments, 24 hours old bacterial colonies were picked and resuspended in 100 µl of PBS. Bacteria were serially diluted 1/20 in conditioned medium (10 µl in 200 µl final, in 96-well flat bottom plates). After incubation for 24 hours, bacterial growth was monitored using a microplate spectrometer (Optima Fluostar, BMG Labtech, Champigny, France) by measuring the optical density (OD) at 595 nm [16]. All the experiments were analysed on these serial dilutions, allowing the selection of the dilution still containing at 24 h bacteria in the growth phase for the control (OD595 usually from 0.3 to 0.5). The corresponding bacterial content in CFU/ml was measured by serial dilution plating on LB agar and colony counting. For an OD of 0.3, the CFU content per ml was 9.9×10^7^ for *E. coli*, 14.0×10^7^ for B2599, 69.5×10^7^ for MSSA, 26.0×10^7^ for CNS.

For the analysis of the bactericidal effect, following 24 hours of bacteria incubation in 150 µl conditioned media the OD was measured (time = 0), then 150 µl of fresh DMEM/F12 containing 1% FCS was added to the dilution that allowed obtaining the lowest bacterial content in both IL4I1 and control conditions. These cultures were used for subsequent OD measures at 7 and 24 hours.

### Mouse Injections

Naïve female C57BL/6 mice (6 to 10 weeks old) were purchased from Charles River. Groups of 3 mice were injected intraperitoneally with a mean of 1.74×10^8^ CFU of MSSA or 20 µg lipopolysaccharide resuspended in 500 µL conditioned PBS from HEK or HEK-mIL4I1. After 24 hours, the mice were sacrificed and the spleens and blood collected. All animal experiments were performed in accordance with local institutional (ComEth) guidelines and with appropriate personal and institutional authorizations. The animals were kept under strict observation during the first hours of the experimental procedure to identify signs of distress. No sign of distress were observed. After 24 h, blood (100 µl) was taken via the retrorbital sinus and mice were sacrificed by cervical dislocation (without anaesthesia) in order to have access to internal organs (spleens) and evaluate bacterial charge.

For the *in vivo* bacterial count assay, dissociated spleen sample supernatants were serially diluted and then inoculated on LB agar plates. Bacterial counts were performed after overnight incubation at 37°C.

### ELISA

Cytokines interferon γ (IFNγ), tumor necrosis factor α (TNF), interleukin 6 (IL-6) and interleukin 10 (IL-10) were detected in mouse plasma samples diluted four fold in sample diluent by ELISA according to the manufacturer’s instructions (BD Biosciences, Le Pont de Claix, France).

### Statistical Analysis

All experiments were performed in duplicate (*in vitro* bacterial count assay) or triplicate (*in vivo* experiments) and were performed 3 to 5 times as stated in the figure legends. Data are given as mean ± SEM. For statistical analysis, the nonparametric Mann-Whitney test was performed using the GraphPad Prism software. *p* values less than 0.05 were considered statistically significant.

## Supporting Information

Figure S1 Quantitative determination of ammonia/ammonium. NH_3_ and NH_4_^+^ were measured in 24 hours Phe-containing conditioned PBS from THP1 and THP1-IL4I1, using an enzyme-based assay. Results from 6 and 5 independent samples, respectively, with mean ± SEM, are shown. *p = 0.03, Mann-Whitney test.
(TIF)

Figure S2 HPLC analysis of Phe, Trp and phenylpyruvate content in THP1 and THP1-IL4I1 conditioned media. Twenty µl of DMEM/F12 media were separated by a mixed mode ion exchange and reverse phase HPLC technique. (**A**) 2 mM of Phe (left), phenylpyruvate (center) or Trp (right) were added to DMEM/F12 to identify the retention times (black arrows). Red arrows indicate the dimethylaminobenzoic acid internal standard (2 mM). (**B**) Representative chromatograms of 24 hours conditioned media: THP1, left; THP1-IL4I1, right. Lower panels are enlargements of the circled areas corresponding to the phenylpyruvate peaks. The first peak corresponds to phenylpyruvate and the second peak to a degradation product. The red dotted line is placed at 100 units on the absorbance scale to allow a better visualization of the difference in peak areas. (**C**) Results expressed as peak area ratio (for phenylpyruvate, the sum of both peaks was considered) are shown for five independent samples, which were analysed in two tests. Left: phenylpyruvate (*p = 0.016, Mann-Whitney test), medium: Phe (not significant), right: Trp (not significant). Note that for phenylpyruvate, the concentrations corresponding to the ratios shown are less than 600 µM.
(TIF)

Figure S3 Cooperative effect of H_2_O_2_ and NH_3_ on bacterial growth inhibition. Bacteria (MSSA) were serially diluted in RPMI 1640 with or without 1 µM to 1 mM isomolar combinations of two of the following products: phenylpyruvate, H_2_O_2_ and NH_3_. After 24 hours, bacterial growth was monitored at an OD of 595 nm. Data are given as mean ± SEM from five independent experiments performed in duplicate. **p<0.01 and *p<0.05, Mann-Whitney test in comparison to RPMI 1640.
(TIF)

Figure S4 Susceptibility of bacteria to catabolite mix in HEPES containing medium. Bacteria were serially diluted in DMEM/F12 with or without isomolar addition of phenylpyruvate, H_2_O_2_ and NH_3_ from 1 µM up to 1 mM. After 24 hours, bacterial growth was monitored at an OD of 595 nm. Data are given as mean ± SEM from five independent experiments performed in duplicate. **p<0.01, Mann-Whitney test in comparison to DMEM/F12.
(TIF)

Figure S5 Potentiation of H_2_O_2_ antibacterial effect via basification of the medium. (**A**) *E. coli* growth is preserved in pH ranging from 6.5 to 9.5. RPMI has a pH of 7.5. The table displays the various additions made to RPMI to obtain pH ranging from 6.5 to 13. *E. coli* was cultured 24 hours in the different RPMI-based media and bacterial growth was monitored at an OD of 595 nm. Data are given as mean ± SEM from 2 to 8 independent experiments performed in duplicate (**B**) *E. coli* becomes sensitive to a non-toxic dose of H_2_O_2_ in basic medium (pH equal or superior to 8.5). *E. coli* was cultured 24 hours in RPMI containing or not NaOH (2.5 or 5 mM) and/or H_2_O_2_ (100 µM). The pH of the media are indicated in the figure. Bacterial growth (OD 595 nm) are given as mean ± SEM from four independent experiments performed in duplicate *p<0.05 and **p<0.01, Mann-Whitney test, according to the bars on the graph.
(TIF)

Figure S6 Cytokines in naïve mice plasma. Interferon-γ (IFNγ), tumor necrosis factor α (TNF), interleukin-6 (IL-6) and interleukin-10 (IL-10) were measured by ELISA in diluted plasma samples from naïve mice. Two to three mice were analyzed. The mean result is indicated by the horizontal bar.
(TIF)

Figure S7 IFNγ production in T cells and NK cells from mice injected with IL4I1 and LPS. Mice were injected i.p. with LPS resuspended in HEK-PBS (n = 3) or in IL4I1-PBS (n = 3). Splenocytes were collected at 24 h and restimulated *in vitro* with PMA and ionomycin. Intracellular IFNγ was measured by flow cytometry in the NK1.1 and the CD3 positive lymphocyte populations. No significant difference was observed in the splenocytes from mice receiving or not IL4I1. The dot-plots show representative results in NK (right) and T cells (left) from one mouse.
(TIF)

Methods S1 (DOC)
