# Supplementary figures and images for: Antibacterial Properties of the Mammalian L-Amino Acid Oxidase IL4I1
Source: PLoS One. 2013 Jan 23;8(1):e54589. doi: 10.1371/journal.pone.0054589 (PMC3552961; doi:10.1371/journal.pone.0054589)

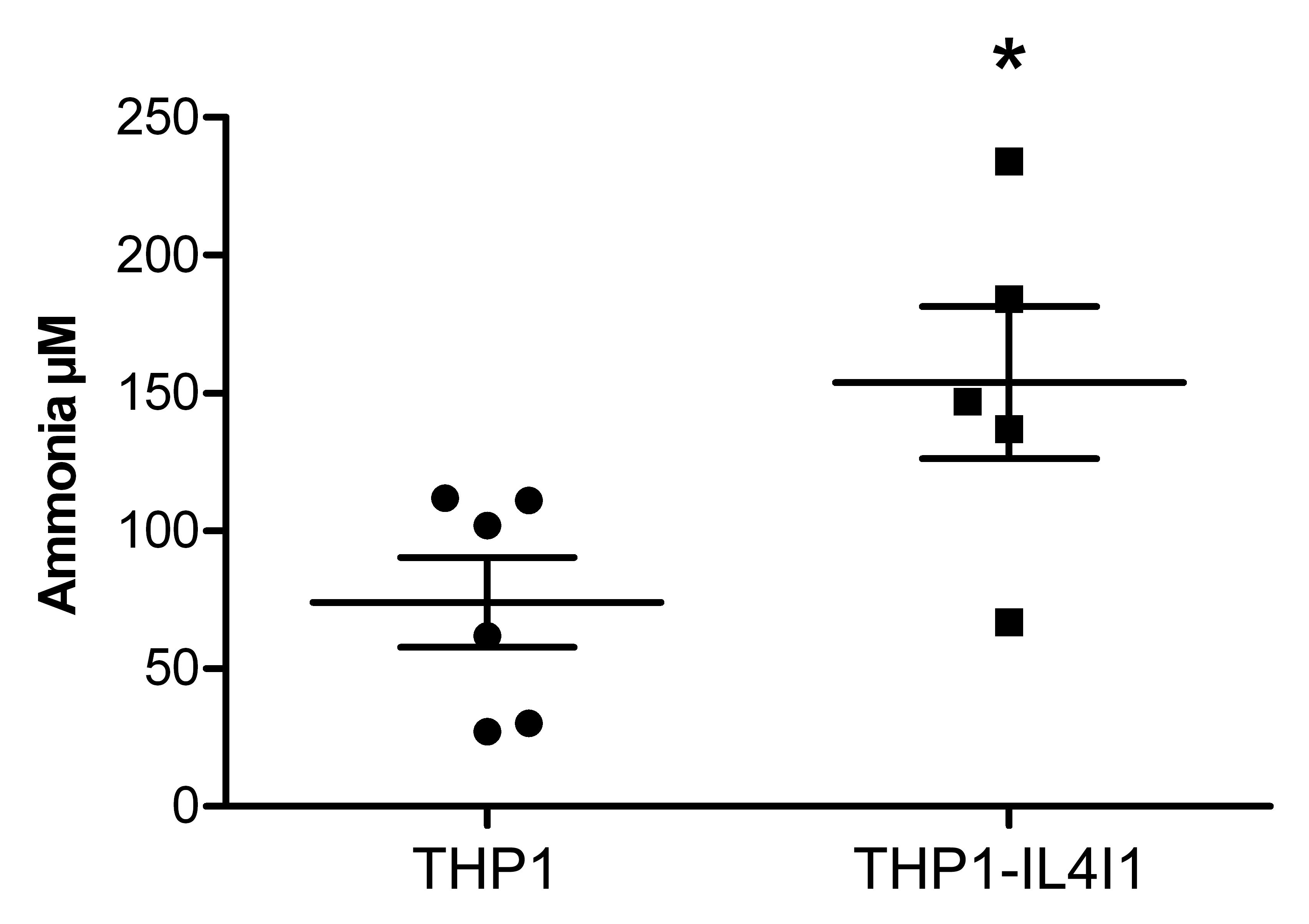

Supplement: Figure S1 — Quantitative determination of ammonia/ammonium. NH3 and NH4 + were measured in 24 hours Phe-containing conditioned PBS from THP1 and THP1-IL4I1, using an enzyme-based assay. Results from 6 and 5 independent samples, respectively, with mean ± SEM, are shown. *p = 0.03, Mann-Whitney test. (TIF) [file pone.0054589.s001.tif]

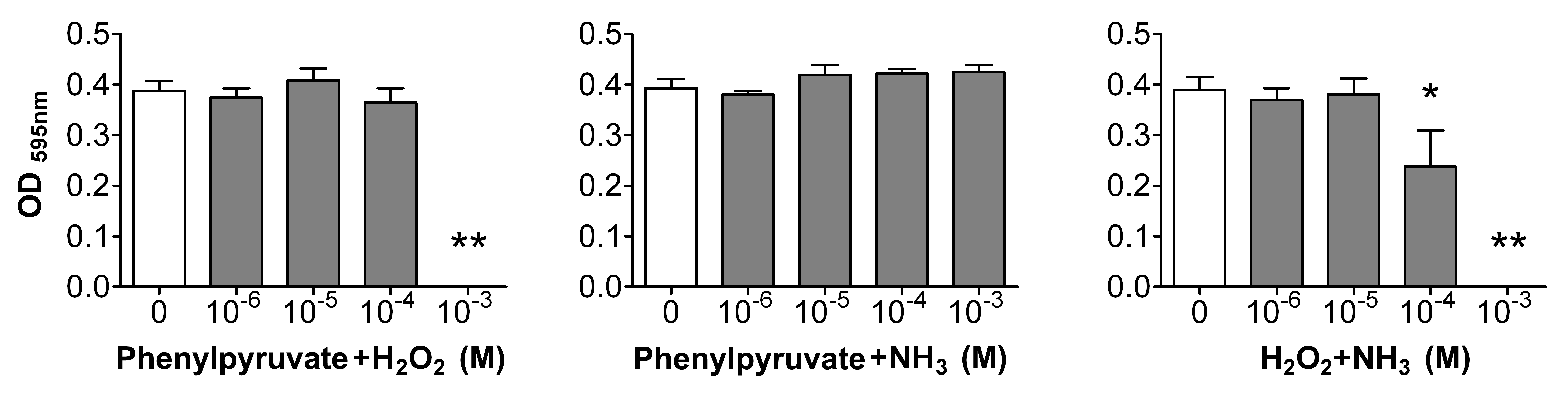

Supplement: Figure S3 — Cooperative effect of H2O2 and NH3 on bacterial growth inhibition. Bacteria (MSSA) were serially diluted in RPMI 1640 with or without 1 µM to 1 mM isomolar combinations of two of the following products: phenylpyruvate, H2O2 and NH3. After 24 hours, bacterial growth was monitored at an OD of 595 nm. Data are given as mean ± SEM from five independent experiments performed in duplicate. **p<0.01 and *p<0.05, Mann-Whitney test in comparison to RPMI 1640. (TIF) [file pone.0054589.s003.tif]

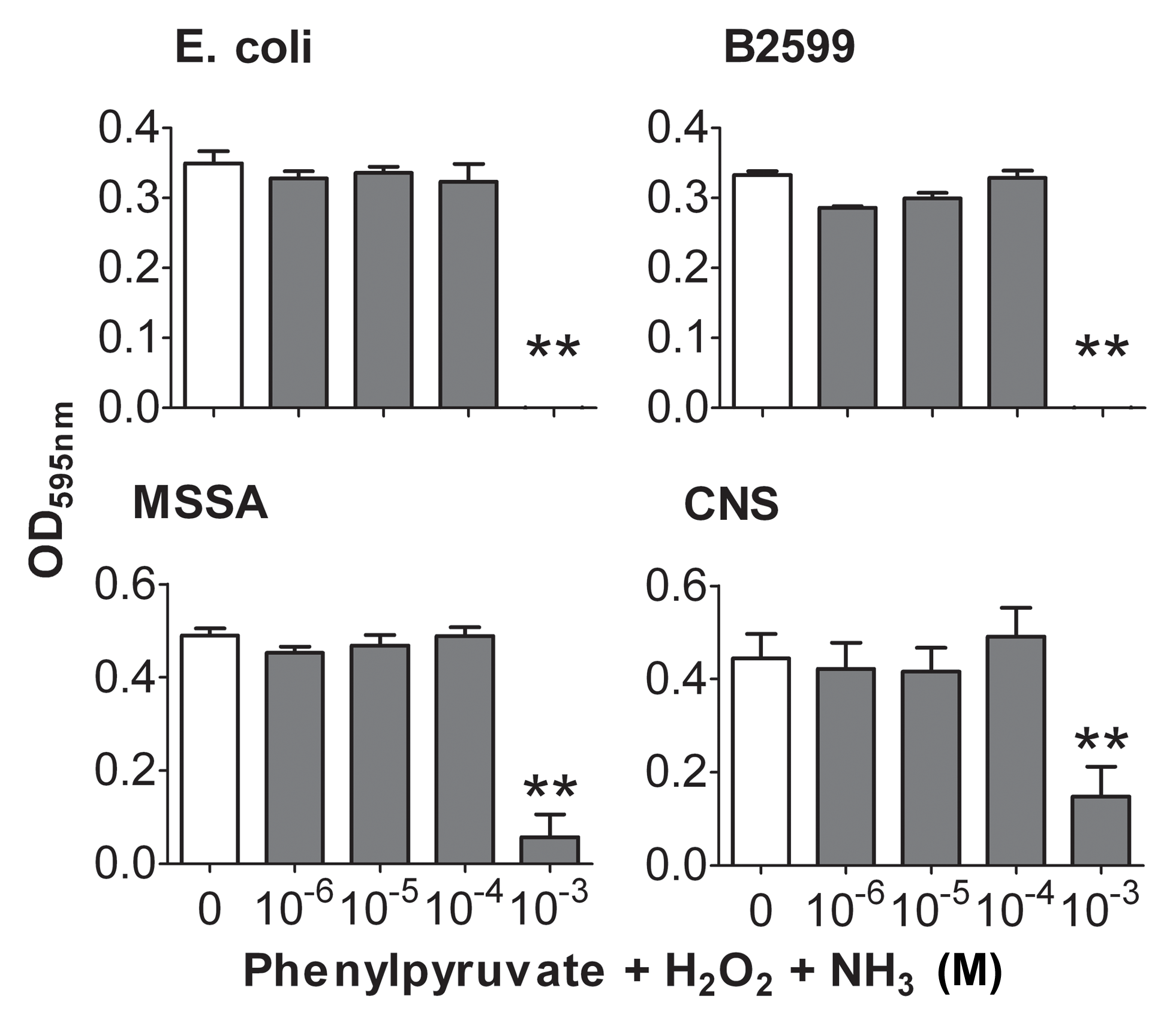

Supplement: Figure S4 — Susceptibility of bacteria to catabolite mix in HEPES containing medium. Bacteria were serially diluted in DMEM/F12 with or without isomolar addition of phenylpyruvate, H2O2 and NH3 from 1 µM up to 1 mM. After 24 hours, bacterial growth was monitored at an OD of 595 nm. Data are given as mean ± SEM from five independent experiments performed in duplicate. **p<0.01, Mann-Whitney test in comparison to DMEM/F12. (TIF) [file pone.0054589.s004.tif]

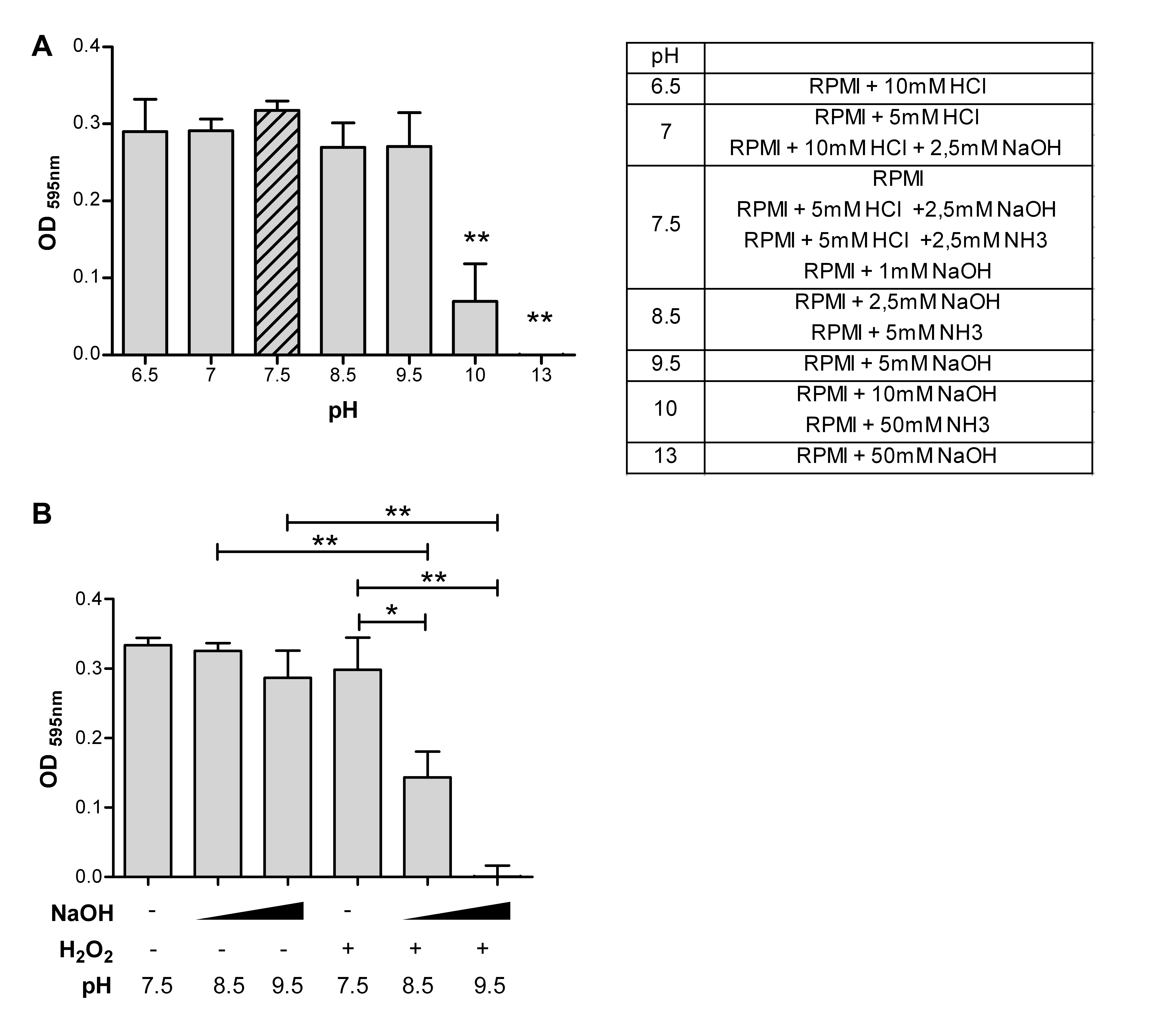

Supplement: Figure S5 — Potentiation of H2O2 antibacterial effect via basification of the medium. (A) E. coli growth is preserved in pH ranging from 6.5 to 9.5. RPMI has a pH of 7.5. The table displays the various additions made to RPMI to obtain pH ranging from 6.5 to 13. E. coli was cultured 24 hours in the different RPMI-based media and bacterial growth was monitored at an OD of 595 nm. Data are given as mean ± SEM from 2 to 8 independent experiments performed in duplicate (B) E. coli becomes sensitive to a non-toxic dose of H2O2 in basic medium (pH equal or superior to 8.5). E. coli was cultured 24 hours in RPMI containing or not NaOH (2.5 or 5 mM) and/or H2O2 (100 µM). The pH of the media are indicated in the figure. Bacterial growth (OD 595 nm) are given as mean ± SEM from four independent experiments performed in duplicate *p<0.05 and **p<0.01, Mann-Whitney test, according to the bars on the graph. (TIF) [file pone.0054589.s005.tif]

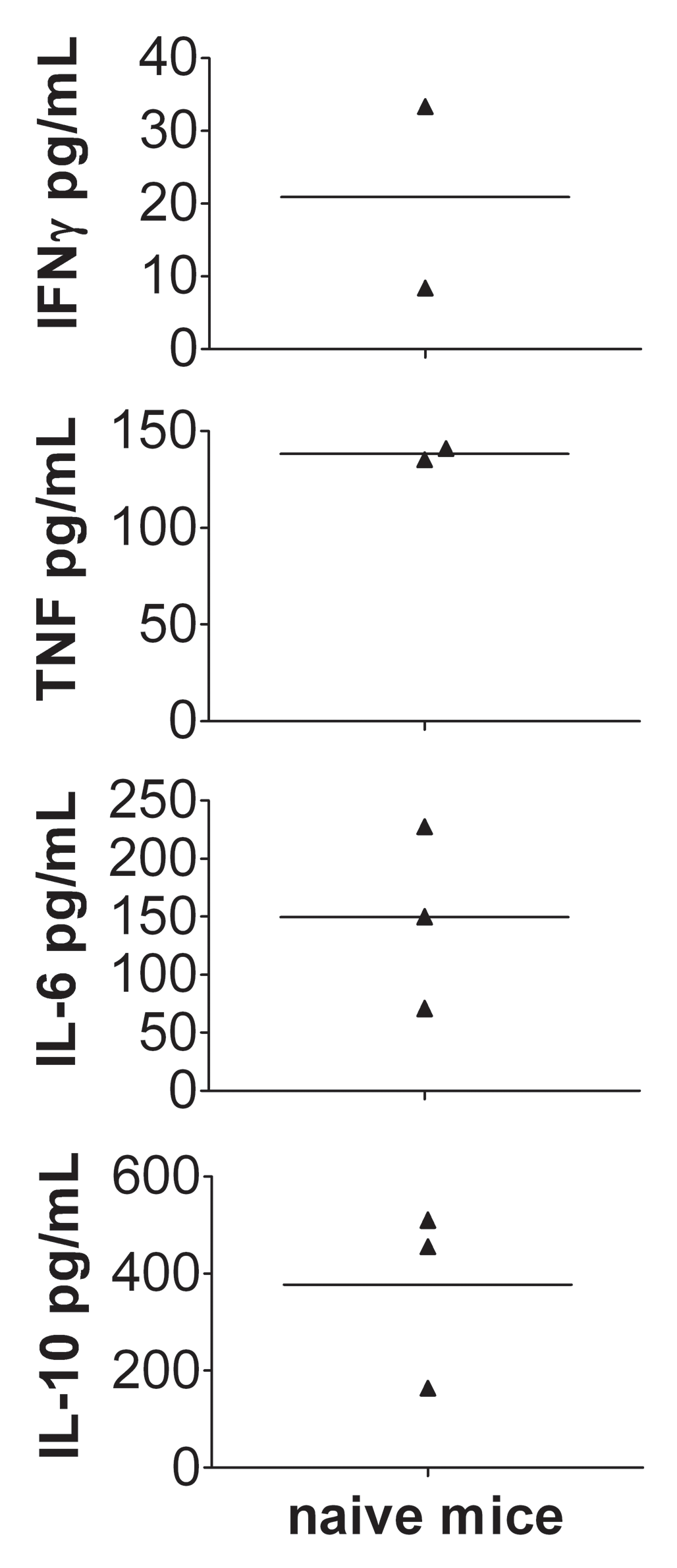

Supplement: Figure S6 — Cytokines in naïve mice plasma. Interferon-γ (IFNγ), tumor necrosis factor α (TNF), interleukin-6 (IL-6) and interleukin-10 (IL-10) were measured by ELISA in diluted plasma samples from naïve mice. Two to three mice were analyzed. The mean result is indicated by the horizontal bar. (TIF) [file pone.0054589.s006.tif]

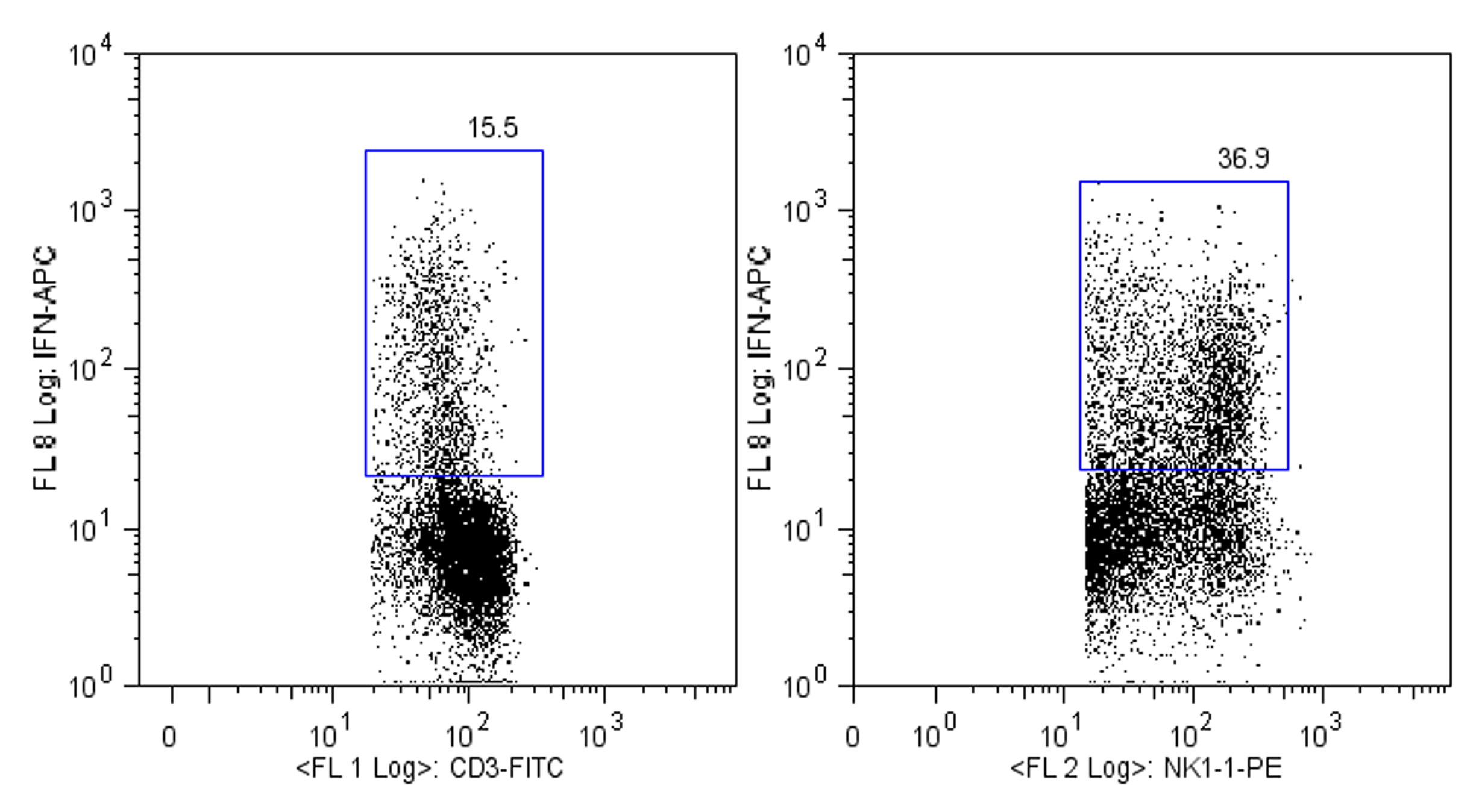

Supplement: Figure S7 — IFNγ production in T cells and NK cells from mice injected with IL4I1 and LPS. Mice were injected i.p. with LPS resuspended in HEK-PBS (n = 3) or in IL4I1-PBS (n = 3). Splenocytes were collected at 24 h and restimulated in vitro with PMA and ionomycin. Intracellular IFNγ was measured by flow cytometry in the NK1.1 and the CD3 positive lymphocyte populations. No significant difference was observed in the splenocytes from mice receiving or not IL4I1. The dot-plots show representative results in NK (right) and T cells (left) from one mouse. (TIF) [file pone.0054589.s007.tif]
