# Supplementary material for: Antibacterial Properties of the Mammalian L-Amino Acid Oxidase IL4I1
Source: PLoS One. 2013 Jan 23;8(1):e54589. doi: 10.1371/journal.pone.0054589 (PMC3552961; doi:10.1371/journal.pone.0054589)
Supplement: Methods S1 — (DOC) [file pone.0054589.s008.doc]

## Supporting Information Methods

**HPLC quantification of Phe, Trp and phenylpyruvate**. HPLC analysis was conducted on a Hitachi Diode array C2455 Elite device equipped with a Lachrom pump L2130 and an L2300 oven using a 20 µl injection loop. The separation was obtained on a mixed-mode Primesep 100 column (150x4.6 mm) packed with 5 µm particles (Sielec, Prospect Hights, Illinois, USA). Samples were separated using a linear gradient of acetonitrile (20 to 60%) in water/tricholoroacetic acid pH 2.5 at a flow rate of 1ml/min at 30°C. Elution of the different products was followed at 208 nm. Dimethylaminobenzoic acid (retention time 16.7 min) was used as an internal standard. Retention times of Phe and Trp were 22.05 and 30.30 minutes, respectively. The two peaks of phenylpyruvate had retention times of 4.03 and 7.53 minutes, respectively.

Twenty-four hours conditioned DMEM/F12 media from THP1 and THP1-IL4I1 cells were filtered through a 0.22 µm disposable unit. Two millimolar internal standard was added and samples precipitated 30 min with 10% trichloracetic acid at 4°C. Precipitates were centrifuged at 10,000 g for 10 min at 4°C and supernatant containing amino acids were kept frozen until injection. The amount of phenylpyruvate, Phe and Trp were quantified as the peak area ratio between each compound and the internal standard, using the EZchrom Elite software (Hitachi).

**Quantitative determination of ammonia/ammonium**. Fluorimetric detection of NH_3_ and NH_4_^+^ was performed on 20 µl of 24 hours conditioned PBS from THP1 and THP1-IL4I1 (10^6^ cells /ml) added with 66 mg/ml of Phe (equivalent to DMEM content) using the EnzyChrom ammonia/ammonium kit (Bioassay system, Hayard, CA, USA). The detection method is based on NADH conversion to NAD^+^ by glutamate dehydrogenase in the presence of NH_3_ and ketoglutarate (detection range 24-1000 µM NH_3_).

**Flow cytometry IFN-γ analysis**

Splenocytes collected from mice receiving LPS in HEK-PBS or IL4I1-PBS were restimulated *in vitro* with PMA (50 ng/ml) and ionomycin (500 ng/ml) for 4h. Brefeldin A (1 µl/ml, Golgi Plug, BD) was added after 1h. Cells were first stained with anti-CD3 FITC and anti-NK1.1 PE (clones 145-2C11 and PK136, respectively, both from e-Biosciences). After fixation and permeabilization (Fix/Perm, e-Biosciences), anti-IFNγ APC (clone XMG1.2, e-Biosciences) was added. At least 150,000 events were acquired in the FS/SS lymphocyte gate, on a Cyan flow cytometer (Beckman-Coulter) and data were analyzed using the Flow-Jo software.
